# Dietary Polyphenols and Periodontitis—A Mini-Review of Literature

**DOI:** 10.3390/molecules23071786

**Published:** 2018-07-20

**Authors:** Arpita Basu, Emily Masek, Jeffrey L. Ebersole

**Affiliations:** 1 Department of Kinesiology and Nutrition Sciences, School of Allied Health Sciences, University of Nevada Las Vegas, 4505 S Maryland Parkway, Las Vegas, NV 89154, USA; masek@unlv.nevada.edu; 2 Department of Biomedical Sciences, School of Dental Medicine, University of Nevada Las Vegas, Las Vegas, NV 89154, USA; jeffrey.ebersole@unlv.edu

**Keywords:** periodontitis, polyphenols, green tea, inflammation, *P. gingivalis*

## Abstract

Periodontitis, which is a chronic infection and disease of the periodontium, is a significant global health burden and is linked to other chronic health conditions such as diabetes and cardiovascular diseases. Dietary polyphenols present in a wide variety of plant-based foods, herbs, and botanicals have been shown to exert antimicrobial, anti-inflammatory, and reduced osteoclast and alveolar bone loss activities in animal models of periodontitis. Polyphenol-containing beverages and foods especially green tea and its active catechin epigallocatechin-3-gallate, cranberries, pomegranates, and fruit and vegetable extracts have reported bacteriostatic/bactericidal activity against microbial species such as *P. gingivalis* and shown total bacterial burden in clinical studies. These polyphenols also exhibit anti-inflammatory and antioxidant effects, which have the potential to impact various biological mechanisms for reducing the initiation and progression of periodontitis. The main objective of this mini-review is to focus on the mechanisms of action of dietary polyphenols in improving the pathophysiology underlying chronic inflammatory diseases like periodontitis based on pre-clinical and clinical models.

## 1. Introduction

Two of the most prevalent forms of oral conditions are dental plaque-induced gingivitis and chronic periodontitis. Based on the National Health and Nutrition Examination Survey (NHANES) (2009–2012), approximately 50% of US adults greater than 30 years old exhibit periodontitis with Hispanics, non-Hispanic Blacks, and aging individuals disproportionately affected by this condition [1]. In this previous cross-sectional study, periodontitis was defined by combining measures of clinical attachment loss (AL) and periodontal probing depth (PD) on all teeth except third molars, which is defined by the American Academy of Periodontology [2]. Based on the Global Burden of Disease 2010 study, severe periodontitis is a significant global health burden and represents the sixth most prevalent condition worldwide. It affects approximately 743 million people worldwide [3] Periodontitis is a localized inflammatory process mediating the destruction of soft and hard periodontal tissues triggered by a complex bacterial biofilm insult. Poor dental behaviors and oral hygiene have been determined to be the primary factors in disease risk and expression and are associated with many dietary and lifestyle factors as well as the concomitant presence of chronic systemic conditions such as diabetes [4]. Based upon the disruption of the oral epithelial barrier, periodontitis is also characterized by systemic inflammatory host responses that may contribute to the higher risk of cardiovascular disease (CVD) among affected patients [5]. Several reports consistently demonstrate a positive correlation between periodontitis and vascular diseases including reports about diabetes [6,7,8,9]. Microbial translocation from periodontal lesions and resulting systemic inflammation are considered common links that underlie these associated conditions with elevated systemic inflammation determined by biomarkers such as the C-reactive protein and pro-inflammatory cytokines and chemokines correlated to periodontitis [10,11]. These observational data identify the need for optimal oral health as an integral component for the prevention and management of chronic conditions such as CVD and diabetes. Dietary nutrients and specific bioactive compounds including polyphenols have emerged as influential factors in the etiology and progression of periodontitis. In recent years, observational studies show a strong correlation between the intake of fruits and vegetables and other antioxidant nutrients with oral health-related quality of life in adults [12,13]. These effects have been mainly attributed to their function in reducing oxidative stress, inflammation, and replacing less healthy dietary choices that promote increased bacterial burden and associated inflammation. Nutritional studies are now recognizing the role of food groups high in vitamin C and other compounds such as the anti-inflammatory omega-3-fatty acids and fiber in decreasing the risks of periodontitis [4,14,15]. While these reports are promising, further research deserves urgent attention given the high prevalence of periodontitis in the US and global population. Given the lack of reviews in this area of research, the primary objective of this focused narrative is to review and discuss the salient findings of reported nutritional studies in recent years. This paper outlines the role of dietary polyphenols in periodontitis.

## 2. Dietary Polyphenols: Link with Chronic Diseases and Periodontitis

Dietary polyphenols from commonly consumed foods and beverages as well as herbs and botanicals are well known for their anti-microbial, antioxidant, and anti-inflammatory functions. Dietary polyphenols can be broadly grouped into many different categories. These categories include tannins, flavonoids, and lignin-carbohydrate complexes (LCC), which have been associated with anti-microbial and anti-inflammatory properties in mechanistic studies [16], as well as those that are commonly consumed as dietary sources. For example, 437 polyphenol compounds were identified in raw and prepared foods in a newly established database for European countries [17]. Among these, the dietary flavonoids and their sub-classes (anthocyanidins, flavones, flavan-3-ols, flavonols, and flavanones) have been widely studied for their associations with chronic diseases including those related to inflammation [18,19,20]. Epidemiological and clinical studies over the last decade have accumulated substantial evidence about the inverse associations of dietary polyphenol intake especially the flavonoids found in fruits, tea, and olives with a risk for several chronic diseases such as diabetes [21], cardiovascular diseases [19], cancer [22], and neurodegenerative diseases [20]. Based on the emerging relationship of these conditions with periodontitis [23,24], studying a potential causal link of dietary polyphenols in lowering the risks of periodontitis is a critical research direction. Green tea, which is a rich source of flavonoids such as catechins, have been shown to exert significant antioxidant and bactericidal activities in small clinical trials when participants consumed green tea supplements [25] or slow release strips of green tea catechins mechanically applied into dental pockets [25]. A recent paper reviewed the role of dietary polyphenols in oral health and identified the anti-viral and anti-inflammatory effects of polyphenols derived from green tea and black tea. The paper also identified licorice root as topical agents that can be applied directly to the oral cavity [16]. Based on the results from various experimental models and clinical studies, designing polyphenol formulations into local delivery applications may present an emerging line of natural therapy for periodontitis and may maximize improved oral health among more populations.

## 3. Dietary Polyphenols and Periodontitis: Cellular Studies

Many in vitro studies have examined the effects of dietary polyphenols on inflammatory markers and pathogenicity of bacteria associated with periodontitis (Table 1). A variety of microorganisms have been detected in dental biofilms and in periodontopathic biofilms. These bacteria include *Porphyromonas gingivalis*, *Tannerella forsythia*, *Treponema denticola*, and *A. actinomycetemcomitans* [26]. However, recent microbiome studies have suggested the contribution of other periodontopathogens [23]. Dietary sources of polyphenols including curcumin, green tea, and pomegranates have been identified for their potential role in treating inflammatory responses of gingival and periodontal diseases. Using a comprehensive in vitro assay, Shahzad et al. screened 48 polyphenolic compounds for their role in inhibiting periodontal pathogens including the major categories of polyphenols such as organic acids (hydroxybenzoic acids, hydroxycinnamic acids, hydroxyphenylacetic acids), flavanols, flavanones, anthocyanins, flavones, isoflavonoids, and phenolics. When applied to bacterial strains implicated in periodontitis, it was observed that the curcumin was the most potent inhibitor of bacterial growth. This was followed by pyrogallol, pyrocatechol, and quercetin [26]. Notably, these polyphenol treatments were demonstrated to selectively target pathogenic biofilm microorganisms especially *P. gingivalis* while sparing normal microbiota members of the dental biofilm such as *Streptococcus mitis* [26]. The viability, proliferation, and biofilm-forming capacity of pathogens associated with periodontitis can be significantly affected by dietary polyphenols. Other commonly consumed dietary sources of polyphenols, such as blueberry extract and tea polyphenols (black and green tea extracts and theaflavins) have also been shown to inhibit biofilm formation and slow bacterial growth in several studies [26,27,28]. The addition of epigallocatechin-3-gallate (EGCG), which is the most abundant and bioactive green tea polyphenol, and green tea extract to a culture of human gingival epithelial cells inhibited the release of several cytokines [29].

Less commonly used herbal extracts such as common sorrel and *Limonium brasiliense* extracts were shown to reduce the adherence of *P. gingivalis* to human gingival fibroblast cells. The mechanism has been explained to be the potential interaction of polyphenols with protein complexes called gingipains [30,31]. Polyphenols have also been demonstrated to exert antioxidant properties and inhibit the release of inflammatory cytokines in vitro [29]. The use of resveratrol, which is a phenolic compound in grapes and wine, reduced nitric oxide expression in a dose-dependent and time-dependent manner in human periodontal ligament cells exposed to *P. gingivalis* [32]. These promising in vitro effects of the polyphenols in altering various responses that could contribute to pathological changes in periodontitis deserve extended investigations using animal models and human clinical studies.

## 4. Dietary Polyphenols and Periodontitis: Animal Studies

A limited number of in vivo studies using rodent models have provided mechanistic data on the role of dietary polyphenols in alleviating features of periodontitis (Table 2). Polyphenol treatment of animal disease models has shown to decrease inflammatory markers and macroscopic damage associated with periodontal disease [33,34,35,36]. Oral intake of EGCG and curcumin was observed to lower circulating levels of inflammatory cytokines known as IL-1β, TNF-α, and IL-17, which are implicated in the inflammation and disease progression of periodontitis [33,34]. Animal studies using St. John’s wort (*Hypericum perforatum*) and green tea extract found that these treatments reduced the extant of bacteria-induced immune cell infiltration into the periodontal tissues, which could help mitigate further inflammatory damage [35,36]. 

Animal studies have also specifically documented the effects of polyphenol treatment on alveolar bone loss. Alveolar bone resorption is a hallmark of periodontitis due to the deleterious effects of inflammatory cytokines on osteoclast numbers, maturation, and function. Osteoclastogenesis is excessively stimulated during the inflammatory process. The resulting dysregulation of bone formation and resorption due to the surplus of active osteoclasts dissolves the mineral matrix of the bone [37]. Oral intake of myricetin, which is a polyphenol derived from many plant foods, was observed to reduce alveolar bone loss in mice by interacting with osteoclast-related genes at doses of 10 μM and 50 μM concentrations [37]. Similar results have also been demonstrated following treatment with mangiferin, which is a polyphenol present in mangos, at 50 mg/kg. This revealed reduced bone loss in mice with decreased levels of IL-6 and IL-1β, which directly interact with the osteoclastogenic pathways and promote the maturation of osteoprogenitor cells into mature osteoclasts [38]. Resveratrol and curcumin treatment also demonstrated amelioration of bone loss in rats using antioxidants and reducing inflammatory cytokines at doses of 10 mg/kg and 100 mg/kg, respectively [34]. 

An important point to consider is the limited physiological relevance of the large polyphenol doses used in cell and animal studies. In most cases, dietary polyphenols have poor bioavailability in humans and undergo rapid metabolism and excretion. For example, feeding studies have shown consumption of 100 g dietary berries lead to concentrations of serum quercetin, which is a common dietary polyphenol that ranges from 15–25 µM in middle-aged adults [39]. Similarly, studies have reported non-detectable or very low levels of curcumin in clinical trials after a large dose intervention (~8 g curcumin/day) [40]. Therefore, while the pre-clinical data are intriguing, further studies are needed at habitual levels of intake in human clinical trials of periodontitis. These actions of dietary polyphenols in animal models of periodontitis provide consistent support for increasing exposure to common sources of dietary polyphenols such as curcumin, fruits like grapes and mangoes, and green tea for preventing and treating this oral disease.

## 5. Dietary Polyphenols and Periodontitis: Human Clinical Studies

For many years, the role of dietary nutrients in oral health has been largely studied from the perspectives of macronutrients such as sugars that can promote dental caries and the growth of microorganisms. Increased sugar and total carbohydrate intake have been associated with an increased risk for developing dental caries and experiencing gingival bleeding [41]. Sugar acts to diminish oral health through fermentative metabolism of many oral bacteria, which results in the release of acidic byproducts that dissolve the mineral content of the teeth [4]. Yet, lactose has been observed to be less cariogenic than other sugars [4]. An analysis of NHANES III (1984–1994) data of young adults demonstrated that a high frequency of consumption of added sugars was associated with a greater prevalence of periodontal disease [42]. Therefore, it is important to consider the influence of dietary sugars for the development and severity of periodontitis even in conjunction with dietary polyphenols.

Clinical studies have also been conducted to determine the potential antimicrobial, antioxidant, and anti-inflammatory properties of dietary polyphenols (Table 3). A clinical study administering freshly-squeezed pomegranate juice as a mouth rinse in subjects without periodontal disease demonstrated significant reductions in the colony-forming units (CFUs) of *Lactobacillus* and *Streptococcus* species [43]. Pomegranates are rich in polyphenols, tannins, ellagic acid, and anthocyanins, which may be implicated in the antimicrobial properties of this mouth rinse. In another clinical study of patients with chronic periodontitis, a gel containing 1% curcumin, which is the bioactive substance found in turmeric, was applied to affected areas in the periodontal pockets and resulted in significant bactericidal effects on *P. gingivalis*, *P. intermedia*, *F. nucleatum*, and *Capnocytophaga* [44]. Other clinical studies have demonstrated beneficial effects of dietary polyphenols on the clinical measurement of periodontal disease including probing depth (PD), gingival index (GI), and clinical attachment level (CAL), which are indicators of periodontitis severity. In one such study, supplementation with a capsule containing selected dehydrated fruits and vegetables was shown to significantly reduce PD compared with placebo pills [45]. In another study, sub-gingival application of a gel containing *Emblica officinalis* or gooseberry extract (10%) showed reductions in PD, an increase in CAL, and improvements in the modified sulcus bleeding index [46]. A similar study of intra-pocket application of a green tea extract gel was demonstrated to decrease PD, GI, and relative CAL (rCAL) in chronic periodontitis patients [47]. Lastly, dietary polyphenols have been shown to possess significant anti-inflammatory and antioxidant properties. In a study of chronic periodontitis, patients were treated with either a green tea dentifrice containing 60% to 90% epigallocatechin or a standard fluoride/triclosan dentifrice. It was found that the green tea treatment significantly increased the activity of glutathione-S-transferase, which is an endogenous antioxidant, and this treatment subsequently decreased the degree of gingival inflammation [48]. 

Another study using dark chocolate, which is rich in cocoa flavonoids, demonstrated that dark chocolate increases total antioxidant capacity and decreases lipid peroxidation and the modified papillary bleeding index when compared with a white chocolate treatment group [49]. While these clinical findings as presented in Table 3 look promising for the management of periodontitis, it is important to consider the lack of characterization and standardization of polyphenol content in foods and beverages that may be associated with differential outcomes in humans. Nevertheless, based on the findings of these clinical studies, increasing oral exposure to dietary polyphenols with a concomitant decrease in sugar intake may be considered a prudent dietary strategy in managing periodontitis.

## 6. Food vs. Purified Polyphenols in Periodontitis

The source of polyphenols must be taken into consideration when interpreting their effects on the outcomes of periodontitis. Commonly consumed dietary polyphenols derived from food and beverages such as green tea [28,48], blueberry [27] and cranberry extracts [50], and pomegranate juice [43] have been shown to inhibit the growth of bacterial biofilms and inflammation and improve clinical outcomes of periodontitis. Green tea is a rich source of several flavonoids especially the gallated catechins [51]. Blueberries are high in anthocyanidins and phenolic acids [52] and cranberries offer proanthocyanidins (PACs) that have been associated with multiple anti-microbial effects [53]. PACs are a class of phenolic compounds that take the form of oligomers or polymers of polyhydroxy flavan-3-ol units such as (+)-catechin and (−)-epicatechin [54]. Pomegranate juice, which is a rich source of tannins, ellagic acid, and anthocyanins, has been shown to exert the highest antioxidant potential among the commonly consumed beverages among US consumers [55]. It also includes observed anti-microbial effects following a mouth rinse [43]. In addition to these sources of food, individual polyphenols such as curcumin [34], epigallocatechin gallate [33], myricetin [37], and mangiferin [38], which represent predominant polyphenols in turmeric, green tea, leafy vegetables, and mangos, respectively, have been mostly shown to decrease inflammation in experimental models of periodontitis. Overall, it appears that the natural combination of polyphenols in extracts from whole foods and beverages exert multi-factorial protective effects compared to isolated polyphenol supplements in periodontitis.

## 7. Conclusions and Recommendations

**Figure 1:**
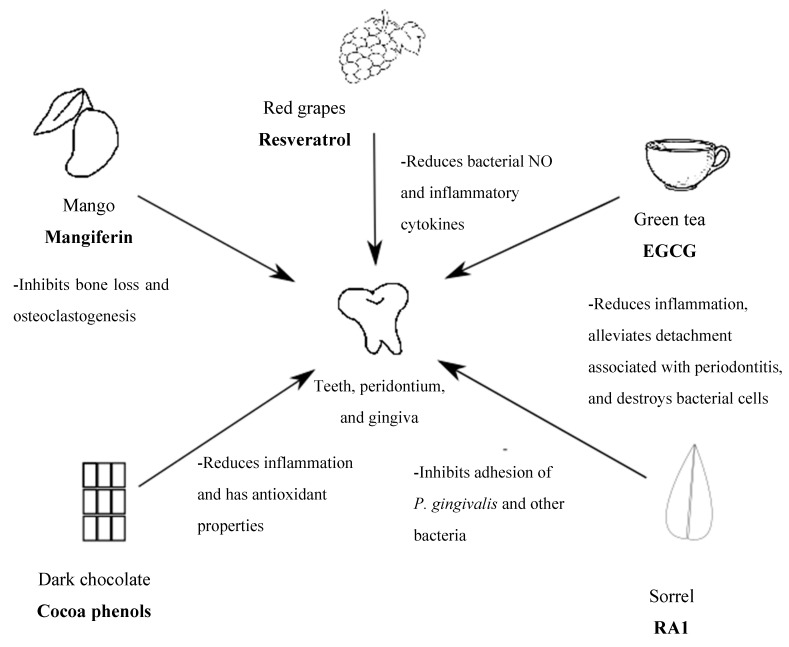
Overview of the role of dietary polyphenols in the management of periodontitis.

Periodontitis is a local inflammatory disease of the oral cavity associated with an increased risk for developing CVD, diabetes, and other chronic diseases, which highlights the urgent need to identify cost-effective population-level strategies for periodontitis prevention and treatment. Current data from cell biology and animal models and human clinical studies have demonstrated that selected dietary polyphenols have important antimicrobial, antioxidant, and anti-inflammatory properties resulting in improved clinical markers in periodontitis (Table 4 and Figure 1,). Dietary polyphenols are derived from a variety of sources such as curcumin in the commonly consumed turmeric and quercetin and catechins in green tea, fruits, and vegetables. Dietary polyphenols have been shown to effectively ameliorate gingival bleeding as well as alveolar bone loss in animals and human clinical studies by suppressing osteoclastogenesis and inhibiting inflammatory cytokines. While data from molecular studies are promising, further research is needed to learn about the effects of polyphenols for prevention and treatment of periodontal disease. The oral cavity, which is the first port of entry for foods andbeverages, is susceptible to their immediate local actions including detrimental effects that contribute to periodontitis. Therefore, the selection of polyphenols at each meal or snack in combination with adequate measures of standard oral hygiene care may play an important role in the prevention of periodontitis as well as other chronic inflammatory conditions that comprise this constellation of co-morbid conditions.

## Figures and Tables

**Table 1:** Polyphenols as antioxidant/anti-inflammatory/antimicrobial agents in periodontitis: experimental cellular models.

Study Design and Model	Polyphenol	Significant Findings	Author, Year
HPLC culture stimulated with LPS of *P. gingivalis*	Resveratrol (25, 50, and 100 µM)	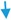 NO expression by *P. gingivalis* in a dose-dependent and time-dependent manner	Rizzo et al., 2012 [32]
*F. nucleatum* cultures in Todd-Hewitt broth	70% ethanolic blueberry extract of varying concentrations (500, 250, 125, 62.5, 31.25, 15.62, and 7.9 μg/mL)	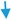 growth of *F. nucleatum* and biofilm formation	Ben Lagha et al., 2015 [27]
Human gingival epithelial cells grown in keratinocyte medium	EGCG (1 and 5 mg/mL) & AC-PCs (25 and 50 mg/mL)	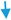 release of several inflammatory cytokines	Lombardo et al., 2015 [29]
*P. gingivalis* culture in sheep blood agar	RA1 (1–100 μg/mL) containing flavan-3-ols, flavonoids, and oligomeric proantho cyanidins	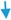 adhesion of *P. gingivalis* to human KB cells due to the specific activity of galloylated oligomeric proanthocyanidins, inhibited gingipain activity, and inhibited *P. gingivalis*-induced hem agglutination	Schmuch et al., 2015 [30]
Bacterial cultures of *S. mitis*, *A actinomycetemcomitans*, *P. gingivalis*, and *F. nucleatum*	Phenols and polyphenols from different classes including hydroxyl acids, flavanols, flavanones, anthocyanins, and phenolics (0.24–2500 μg/mL, depending on the compound)	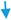 planktonic growth, mostly by curcumin which was followed by pyrogallol, pyrocatechol, and quercetin	Shahzad et al., 2015 [26]
Normal human fibroblasts incubated with HSA, G-HSA, or *P. gingivalis* LPS	Cranberry high molecular weight NDM (10–50 μg/mL with HSA or G-HSA alone; 50 or 100 μg/mL with HSA, G-HSA, and LPS)	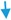 production of IL-6 and MMP-3	Tipton et al., 2016 [50]
*P. gingivalis* strain cultivated in agar medium and broth	*L. brasiliense* extract in water/acetone solution (50–500 μg/mL)	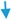 adhesion of *P. gingivalis* to human KB cells and the activity of Arg-gingipain	De Oliveira et al., 2017 [31]
*F. nucleatum* in Todd-Hewitt broth	Green tea (20 mg), black tea (10 mg), and theaflavins (20 mg) in solution	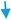 biofilm formation	Ben Lagha et al., 2017 [28]

AC-PC, A-type cranberry proanthocyanidins, EGCG, epigallocatechin gallate, G-HSA, glycated human serum albumin, HSA, human serum albumin, HPLC, human periodontal ligament cell, IL-6, interleukin-6; LPS, lipopolysaccharide, MMP-3, matrix metalloproteinase-3, NDM, non-dialyzable material, NO, nitric oxide; RA1, *Rumex acetosa* (common sorrel) extract. Symbol: downward arrow, decrease.

**Table 2:** Polyphenols as antioxidant/anti-inflammatory/antimicrobial agents in periodontitis: experimental animal models.

Study Design and Model	Polyphenol	Significant Findings	Author, Year
Adult male Sprague-Dawley rats	*Hypericum perforatum* extract (2 mg/kg/day) administered orally	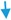 gingivomucosal tissue injury, alveolar bone loss, & expression of NF-κB p65	Paterniti et al., 2010 [35]
*E. coli*-induced periodontitis in Lewis rats	Sunphenon BG blend (91.3% polyphenols) administered topically to affected periodontal areas	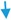 periodontal detachment and bone resorption	Yoshinaga et al., 2014 [36]
*P. gingivalis* induced periodontitis in BALB/c mice	EGCG (0.02%) or vehicle (distilled water) in drinking water	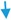 inflammation e.g., IL-17, IL-1β vs. vehicle	Cai et al., 2015 [33]
Ligation-induced periodontitis in Wistar rats	Hawthorn (*Crataeus orientalis* M Bieber) extract (100 mg/kg) vs saline administered orogastrically	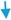 osteoclast activity and subsequently ameliorated alveolar bone loss induced by periodontitis	Hatipoğlu et al., 2015 [55]
C57BL/J6 ovariectomized female mice	Low or high dose (2 or 5 mg/kg/day) of myricetin, which is a polyphenol derived from fruits and vegetables, administered intraperitoneally vs. placebo	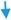 alveolar bone loss by inhibiting osteoclastogenesis induced by periodontitis	Huang et al., 2016 [37]
Wistar rats	Curcumin (100 mg/kg), resveratrol (10 mg/kg), curcumin + resveratrol or resveratrol alone administered orogastrically	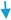 gingival IL-1β in curcumin+resveratrol	Corrêa et al., 2017 [34]
*P. gingivalis* induced periodontitis in male C57BL/6J wild-type mice	Mangiferin (50 mg/kg) oral application	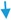 TNF-α production, phosphorylation in the NF-κB and JK-1 signal pathways, and alveolar bone loss	Li et al., 2017 [38]

EGCG, epigallocatechin gallate, *Hypericum perforatum*, St. John’s Wart; IL, interleukin, JK-1, Janus kinase-1, NF-κB, nuclear factor-κB, Sunphenon BG, green tea extract, TNF-α, tumor necrosis factor-alpha. Symbol: downward arrow, decrease.

**Table 3:** Polyphenols as antioxidant/anti-inflammatory/antimicrobial agents in periodontitis: clinical studies.

Study Design and Model	Polyphenol	Significant Findings	Author, Year
Pre-post intervention, healthy volunteers (*n =* 30; age 25–30 years)	Pomegranate juice (30 mL) mouth rinse for 2 min	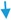 CFUs of both *Streptococci* and *Lactobacillus* spp.	Kote et al., 2011 [43]
Crossover RCT, patients with chronic periodontitis (*n =* 60, age 30–60 years)	Daily oral intake of 6 FV capsules, 6 FVB capsules, or placebo capsules for 2 months	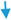 PPD in FV compared with placebo	Chapple et al., 2012 [45]
Patients with chronic periodontitis (*n =* 30; age 38.9–10.67 years)	Sustained-release green tea extract gel (1%) containing ECGC administered once	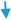 GI, PD, and rCAL	Chava & Vedula, 2013 [47]
Patients with chronic periodontitis (*n =* 25; age 21–45 years)	1% curcumin gel inserted into periodontal pockets with blunt syringe at intervals of 1, 3, and 6 months following the start of the study	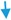 Bacterial counts of *P. gingivalis*, *P. intermedia*, *F. nucleatum*, and *Capnocytophaga* spp.	Bhatia et al., 2014 [44]
Crossover RCT; patients with moderate chronic periodontitis (*n =* 40, age 30–50 years)	Oral intake 3 times a day for 4 weeks of 30 g dark chocolate with 78% cacao (containing flavonoids such as catechin and procyanidins) or white chocolate placebo *w*/*o* cacao	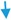 MPBI and lipid peroxidation	Roodgaryan et al., 2015 [49]
Patients treated with root planning and scaling (*n =* 40, age ≥ 30 years)	10% *E. officinalis* extract gel administered subgingivally once and parameters measured 2 and 3 months post-treatment	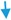 Inflammation, mSBI and PPD	Grover et al., 2016 [46]
Patients with mild to moderate periodontitis (*n =* 30, age 18–60 years)	Green tea extract dentifrice with 60–90% EGCG or placebo dentifrice with fluoride and triclosan, brushed onto teeth for 2–5 min daily for 4 weeks	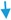 gingival inflammation	Hrishi et al., 2016 [48]

CAL, clinical attachment level, CFUs, colony-forming units, EGCG, epigallocatechin gallate; FV, fruit/vegetable-containing capsules, FVB, fruit/vegetable/berry-containing capsules, GI, gingival index, GST, glutathione-S-transferase, MPBI, modified papillary bleeding index, mSBI, modified sulcus bleeding index, PD, probing depth, PPD, probing pocket depth, rCAL, relative clinical attachment level, RCT, randomized clinical trial, TAC, total antioxidant capacity. Symbol: downward arrow, decrease.

**Table 4:** Summary of mechanisms of action of dietary polyphenols in periodontitis.

Polyphenol	Mechanisms of Action
Resveratrol	Reduces NO expression by *P. gingivalis* bacteria by inhibiting inflammatory cytokines and improving viability of affected HPLCs [32]
Blueberry flavonoids, phenolic acids, and procyanidins e.g., chlorogenic acid, ellagic acid, quercetin, anthocyanins, and proanthocyanidins	Reduce bacterial growth and biofilm formation via antibacterial, inhibitory effects against Gram-negative bacteria such as *F. nucleatum* [26,27,30]
EGCG	Inhibits release of inflammatory cytokines (IL-17, IL-1β) by modulating gene expression pathways (e.g., NF-κB), and decreasing inflammation/oxidation by increasing the activity of GST [33,48]
RA1	Inhibit adhesion of bacteria through a specific activity of galloylated oligomeric proanthocyanidins [30]
Curcumin	Inhibits planktonic growth by decreasing metabolic activity of bacterial species [44]
Curcumin + Resveratrol	Reduces gingival IL-1β and inhibits NF-κB, which lowers proteasome activity and resulting cell damage and inflammation [34]
Pyrogallol	Inhibits planktonic growth by reducing biomass of planktonic films [26]
Pyrocatechol	Inhibits planktonic growth by reducing biomass of planktonic films [26]
Quercetin	Inhibits planktonic growth by reducing biomass of planktonic films [26]
Cranberry flavonoids and proanthocyanidins	Inhibit IL-6 production and MMP-3 by suppressing the NF-κB and MAPK/AP-1 signaling pathways [50]
*L. brasiliense* flavan-3-ols and proanthocyanidins	Reduce adhesion of *P. gingivalis* to human KB cells by inhibiting Arg-gingipain activity [30,31]
Tea polyphenols e.g., theaflavins	Inhibit biofilm formation and adhesion of pathogens to the oral mucosa likely by binding to receptors in the bacterial cell wall [27,28]
*Hypericum perforatum* flavonoids and phenolic acids	Inhibits inflammatory cytokine production by suppressing NF-κB p65 pathway and reducing NO expression by pathogenic bacteria through the suppression of the iNOS system [35]
Myricetin	Reduces alveolar bone loss by inhibiting osteoclastogenesis [37]
Mangiferin	Suppresses TNF-alpha production and inhibits phosphorylation of NF-κB and JK-1 pathways, which inhibits production of inflammatory cytokines and alleviates tissue injury [38]
Pomegranate phenolic compounds	Reduces number of pathogenic *Streptococci* and *Lactobacilli* pathogens and inhibits the formation of colony units [43]
Cacao flavonoids	Decrease lipid peroxidation and improve gingival bleeding [49]
*E. officinalis* flavonoids, phenols, and tannins	May reduce inflammation by suppressing the action of histamine, serotonin, prostaglandins, and other inflammatory mediators [46]

AP-1, activator protein-1, EGCG, epigallocatechin gallate, GST, glutathione-S-transferase, HPLC, human periodontal ligament cell, IL-1β, interleukin-1β, IL-6, interleukin-6, IL-17, interleukin-17, iNOS, inducible nitric oxide synthase, JK-1, Janus-kinase 1 pathway, MAPK, mitogen-activated protein kinases, MMP-3, matrix metalloproteinase-3, NF-κB, nuclear factor-κB, NO, nitric oxide, RA1, *rumex acetosa* extract (polyphenol), TNF-alpha, tumor necrosis factor-alpha.
